# A critical courier role of volatile oils from Dalbergia odorifera for cardiac protection *in vivo* by QiShenYiQi

**DOI:** 10.1038/s41598-017-07659-x

**Published:** 2017-08-04

**Authors:** Jiahui Yu, Wen Zhang, Yiqian Zhang, Yadong Wang, Boli Zhang, Guanwei Fan, Yan Zhu

**Affiliations:** 1 Tianjin State Key Laboratory of Modern Chinese Medicine, Tianjin University of Traditional Chinese Medicine, Tianjin, China; 2 Research and Development Center of CM, Tianjin International Joint Academy of Biotechnology & Medicine, Tianjin, China; 3 State Key Laboratory of Core Technology in Innovative Chinese Medicine, Tianjin Tasly Holding Group Co., Ltd., Tianjin, China; 4 State Key Laboratory of Natural Medicines, China Pharmaceutical University, Nanjing, China; 5 First Teaching Hospital of Tianjin University of Traditional Chinese Medicine, Tianjin, China

## Abstract

Component-based Chinese medicine (CCM) is derived from traditional Chinese medicine but produced with modern pharmaceutical standard and clearer clinical indications. However, it still faces challenges of defining individual component contribution in the complex formula. Using QiShenYiQi (QSYQ) as a model CCM, we investigated the role of Dalbergia odorifera (DO), an herbal component, in preventing myocardial damage. We showed that *in vitro*, QSYQ exerted considerable protective activities on cardiomyocytes from H_2_O_2_-induced mitochondrial dysfunction with or without DO. However, in isolated rat hearts, myocardial protection by QSYQ was significantly weakened without DO. In everted gut sac model, DO significantly enhanced absorption of the major QSYQ ingredients in different regions of rat intestine. Finally, in *in vivo* mouse model of doxorubicin (DOX)-induced myocardial damage, only QSYQ, but not QiShenYiQi without DO (QSYQ-DO), exerted a full protection. Taken together, our results showed that instead of directly contributing to the myocardial protection, Dalbergia odorifera facilitates the major active ingredients absorption and increases their efficacy, eventually enhancing the *in vivo* potency of QSYQ. These findings may shed new lights on our understanding of the prescription compatibility theory, as well as the impacts of “courier herbs” in component-based Chinese medicine.

## Introduction

Cardiovascular disease, especially ischemic heart disease, remains a leading cause of death globally. Because of the complex nature of their etiologies involving genetic and environmental factors, treating cardiovascular diseases with conventional single-targeting drugs has met limitations and therefore, safer and more efficacious therapies are still in great demand. A current trend is drug combinations that include two or more individual drugs into a “Polypill”^1^. For acute myocardial infarction (MI), the use of fixed-dose combinations (FDC) has been shown to improve treatment adherence and risk factor control. For example, in the FOCUS (Fixed Dose Combination Drug (Polypill) for Secondary Cardiovascular Prevention) trial, compared with the 3 drugs given separately, the use of a polypill strategy met the primary endpoint for adherence for secondary prevention following an acute MI^2^.

Chinese medicine (CM) is efficacious in treating various diseases such as ischemic heart disease over a long time and have accumulated thousands of herbal formulas in clinical practice^3, 4^. CM prescription is usually composed of several medicinal herbs, are typical representative of poly-medicines. One essential feature of CM prescription is that the participating herbs are assigned to specific roles of “sovereign-minister-assistant-courier” according to the compatibility theory and contributes differently in prescription^5^. However, while the empiric based CM therapies have been proven clinically effective, the underlying mechanisms for the combined targets remain mysteries.

However, their vast mount of unknown chemical ingredients has posed challenges for the further applications outside China. There is a proposed new concept of component-based Chinese medicine (CCM)^6^, which represents a type of modern Chinese medicine made from standard components guided by the compatibility theory and principles of CM. A possible underlying mechanism of CCMs is multi-components for multiple targets/pathways, which has been illustrated to be especially effective in treating complex diseases^7^. Among which, contribution by the courier herb is always neglected since it usually contributes a small proportion in formulas. Intriguingly, courier herb has been elucidated to be indispensable and play important role in the clinical treatment of promyelocytic leukemia by Chinese medical formula Realgar-Indigo naturalis^8^. Nevertheless, the efficacies and contributions of courier herb have not been fully understood and demonstrated pharmacologically till now.

QiShenYiQi (QSYQ) is a CCM approved by the State Food and Drug Administration of China in 2003. It has been widely used for treating coronary heart disease, angina pectoris and cardiac dysfunction^9^. Pharmacological researches have reported that it improves myocardial function, attenuates cardiac hypertrophy and myocardial fibrosis, inhibites platelet aggregation^10–13^. We have previously shown that QSYQ is also capable of cardioprotection against ischemia/reperfusion injury^14^. Besides, QSYQ is considered to be a typical representative CCM that composed of extracts from Astragalus membranaceus, Salvia miltiorrhiza and Panax notoginseng, as well as volatile oils from Dalbergia odorifera (DO). In QSYQ formula, Astragalus serves as the sovereign, Salvia miltiorrhiza serves as the minister, Panax notoginseng serves as the assistant and Dalbergia odorifera serves as the courier^15^ and therefore, the compatibility theory might be best exemplified by QSYQ formula^16, 17^.

Dalbergia odorifera, the courier herb in QSYQ, has been shown to take effects on antithrombosis, antiplatelet aggregation, antioxidant and anti-inflammation, when applied in cardiovascular disease patients for effective blood circulation and pains relieving^18^. On the other hand, earlier studies suggesting that Dalbergia odorifera may have increase absorption for other active components. For example, it was reported that plasma concentration and the area under curve of tanshinol is significantly increased after administration of Salvia miltiorrhiza coupled with Dalbergia odorifera^19^, but there is no prove in either compound prescriptions. Furthermore, molecular network and pathway analyses suggested that among four component herbs of QSYQ, Astragalus, Salvia miltiorrhiza and Panax notoginseng were much more potent than Dalbergia odorifera for treating AMI^15^.

Based on existing researches and the fact that QSYQ contained only the DO volatile oils that have strong liposolubility, we purposed that instead of exerting pharmacological effect itself, the DO volatile oils might play a more crucial role in facilitating other active compounds absorption and consequently pharmacologically more potent. Therefore, in this study, we carried out series studies using QSYQ with or without DO volatile oils to investigate the efficacies and contributions of this “courier” component in compatibility theory.

## Results

### Chemical profiles of major ingredients in QSYQ were identical with or without DO volatile oils

**Figure 1:**
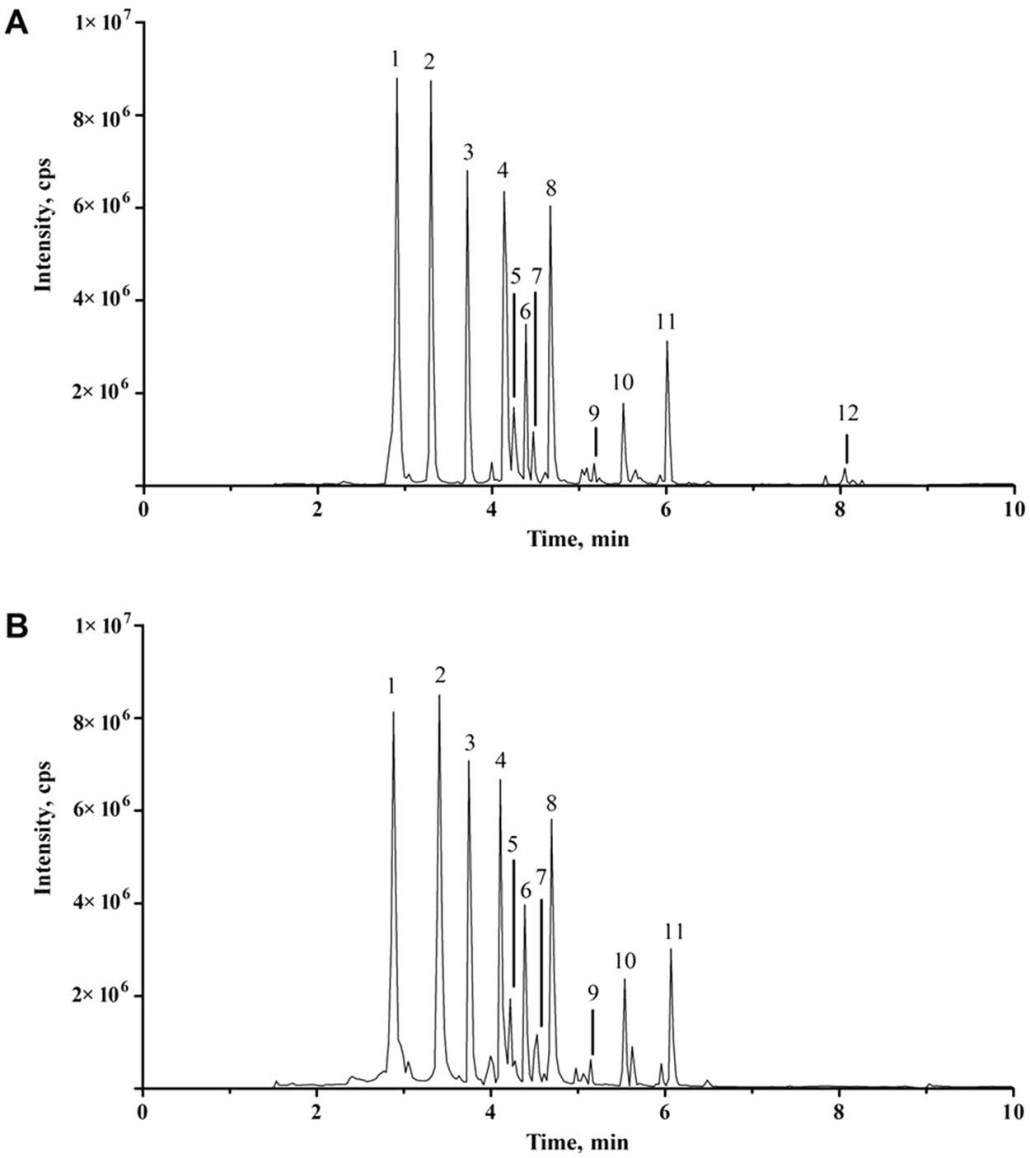
UFLC-MS chromatograms under MRM mode of predominant ingredients in QSYQ (**A**) and QSYQ-DO (**B**). Peak label: 1. Tanshinol, 2. Protocatechuic aldehyde, 3. Calycosin-7-O-β-D-glycoside, 4. Rosmarinic acid, 5. Ononin, 6. Salvianolic acid B, 7. Chloramphenicol (internal standard in negative mode), 8. Calycosin, 9. Astragaloside IV, 10. Formononetin, 11. Diazepam (internal standard in positive mode), and 12. Nerolidol.

To determine the contribution of DO volatile oils in QSYQ formula, chemical profiles of QSYQ with or without DO volatile oils were compared. Ten major chemical ingredients in QSYQ were accurately determined using ultra performance liquid chromatography-mass spectrum (UFLC-MS) both in QSYQ and QSYQ-DO. Chloramphenicol and diazepam were used as internal standard compounds for negative mode and positive mode, respectively. As shown in the chromatograms in Fig. 1, tanshinol (TSL), protocatechuic aldehyde (PCA), calycosin-7-O-β-D-glycoside (CG), rosmarinic acid (RA), ononin (ON), salvianolic acid B (SAB), calycosin (CS), astragaloside IV (AIV) and formononetin (FOR) matched well with previously reported QSYQ components and were identical in QSYQ and QSYQ-DO, except that nerolidol being the marker compound from DO volatile oils in QSYQ.

### Both QSYQ and QSYQ-DO attenuated H_2_O_2_-induced injury in cultured H9c2 cells

**Figure 2:**
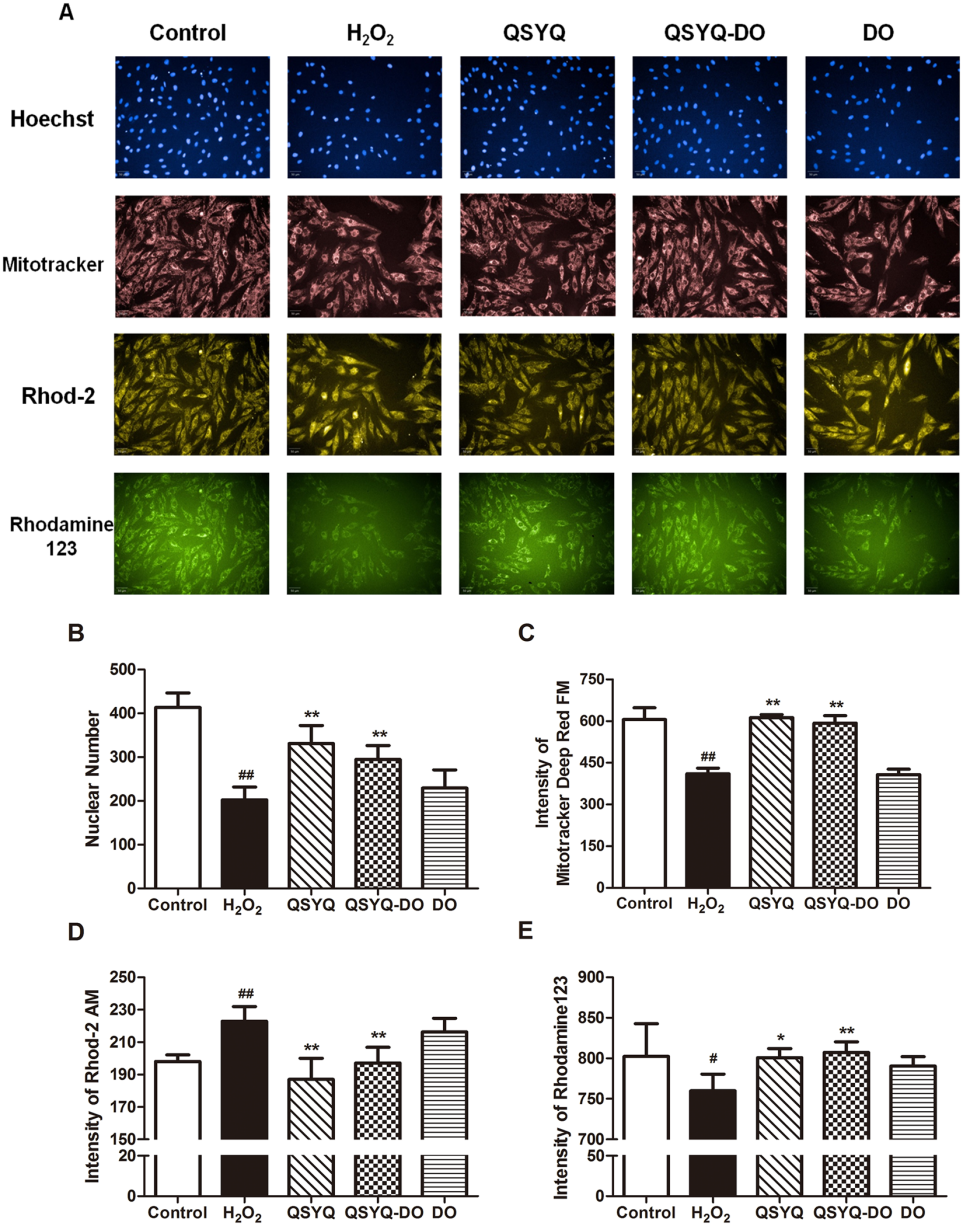
Effects of QSYQ and QSYQ-DO on mitochondrial structure and function against H_2_O_2_ injury. H9c2 cardiomyocytes were treated with QSYQ, QSYQ-DO or DO volatile oils at a concentration of 0.2 mg/mL for 24 h and then exposed to 200 μM H_2_O_2_ for 2 h. (**A**) Representative images of mitochondrial structure and function were captured in Operetta High Content Imaging System. Fluorecent stains: Hoechst for nuclei and cell numbers (blue); Mitotracker for mitochondrial mass (red); Rhod-2 AM for cytosolic Ca^2+^ (yellow) and Rhodamine 123 for ΔΨ_m_ (green). **(B–E)** Quantitative analyses of fluorescence intensities in (**A**). Data are expressed as the mean ± SD, n = 3. ^#^p < 0.05, ^##^p < 0.01 *vs*. Control group. *p < 0.05, **p < 0.01 *vs*. H_2_O_2_ group.

The proper dose in the *in vitro* experiments was first determined. We found that QSYQ at 0.05–0.5 mg/mL did not significantly affect cells viability with an IC_50_ = 1.03 mg/mL (Supplementary Fig. S1A,B). Therefore, 0.05–1 mg/mL QSYQ concentrations were chosen for further investigation. Exposure of H9c2 cells to H_2_O_2_ (200 μM, 2 h) led to a decrease in cell viability whereas treatment with 0.1–0.8 mg/mL QSYQ restored survival of cardiomyocytes (Supplementary Fig. S1C). Therefore, a final concentration of 0.2 mg/mL was chosen for *in vitro* functional studies. In a multi-parameter cell imaging assay, H_2_O_2_ treatment caused reduced cell number (stained by Hoechst 33342) compared to the Control group. However, the reduced nuclear numbers were significantly alleviated with either QSYQ or QSYQ-DO treatment (Fig. 2A,B).

### Both QSYQ and QSYQ-DO alleviated mitochondrial dysfunction in H_2_O_2_-induced injury in H9c2 cells

Since it was known that H_2_O_2_ induced extensive mitochondrial and cell injuries in H9c2 cells, we tested a number of mitochondrial functions, including mitochondrial mass, cytosolic Ca^2+^ and mitochondrial membrane potential (ΔΨ_m_) with fluorescent probes Mito Tracker Deep Red FM, Rhod-2AM and Rhodamine 123 in H_2_O_2_-treated H9c2 cells. As shown in Fig. 2, H_2_O_2_ treatment led to loss of mitochondrial mass and ΔΨ_m_, as well as a cytosolic Ca^2+^ overload. QSYQ and QSYQ-DO treatment significantly prevented the loss of mitochondrial mass (Fig. 2A,C), the decreased ΔΨ_m_ (Fig. 2A,E) and cytosolic Ca^2+^ overload (Fig. 2A,E). On the other hand, DO volatile oils treatment showed only neglectable protective effect on mitochondrial dysfunction. These data indicated that both QSYQ and QSYQ-DO prevented the degree of mitochondrial damage in similar extent and QSYQ-mediated mitoprotection is one of the mechanisms against H_2_O_2_ induced injury, whereas the DO volatile oils in QSYQ does not play a role in these effects.

### DO volatile oils enhanced protective effect for ischemia/reperfusion (I/R) injury in isolated rat hearts

**Figure 3:**
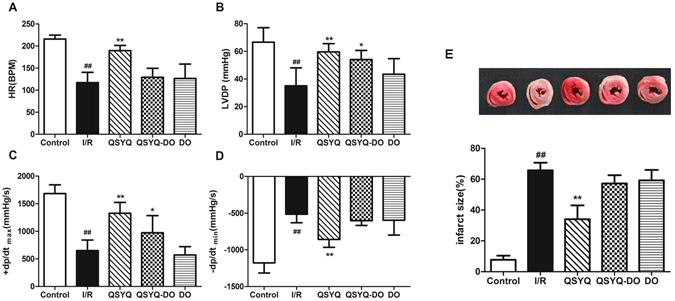
Effects of QSYQ and QYSQ-DO on cardiac function and myocardial infarction size in isolated rat hearts. The hearts underwent 20 min of stabilization, 30 min of ischemia, and 60 min of reperfusion. Drugs were administered before ischemia for 10 min. Cardiac functions were determined by Powerlab data acquisition system. Infarct size was evaluated by TTC staining. (**A**) Heart rate (HR). (**B**) Left ventricular developed pressure (LVDP). (**C** and **D**) Maximal and minimum rate of pressure development (±dp/dt max), respectively. **(E)** Infarct size (%). All parameters were measured at the end of the reperfusion period. Data are expressed as mean ± SD, n = 4. ^##^p < 0.01 *vs*. Control group. *p < 0.05, **p < 0.01 *vs*. I/R group.

We next examined *ex vivo* roles of QSYQ with or without DO volatile oils for I/R injury in isolated rat hearts. The analysis of hemodynamic parameters on cardiac function was shown in Fig. 3. Compared to the Control group, HR, LVDP and ± dp/dt_max_ at the end of the reperfusion period were significantly decreased in I/R group. Pretreatment with QSYQ resulted in improved cardiac function after I/R, with a significant increase in HR, LVDP, and ± dp/dt_max_ compared to the I/R group. However, QSYQ-DO or DO groups did not show, or in a much less extent, such improvement. These data suggest that QSYQ could protect cardiac function against I/R injury more efficiently than those of QSYQ-DO or DO alone. Furthermore, the infarct size of hearts in the QSYQ groups averaged to 34.00 ± 9.02%, which was reduced significantly from the I/R group (66.25 ± 5.06%) whereas there were no significant difference between I/R group and the QSYQ-DO or DO pretreatment groups (Fig. 3E). These data suggest that in contrary to the *in vitro* setting where both QSYQ and QSYQ-DO protected H9c2 cells against H_2_O_2_ injury, QSYQ could protect isolated rat hearts from I/R injury only when in the presence of DO volatile oils.

### Volatile oils from DO facilitated the absorption of major QSYQ components in everted gut sac model

**Figure 4:**
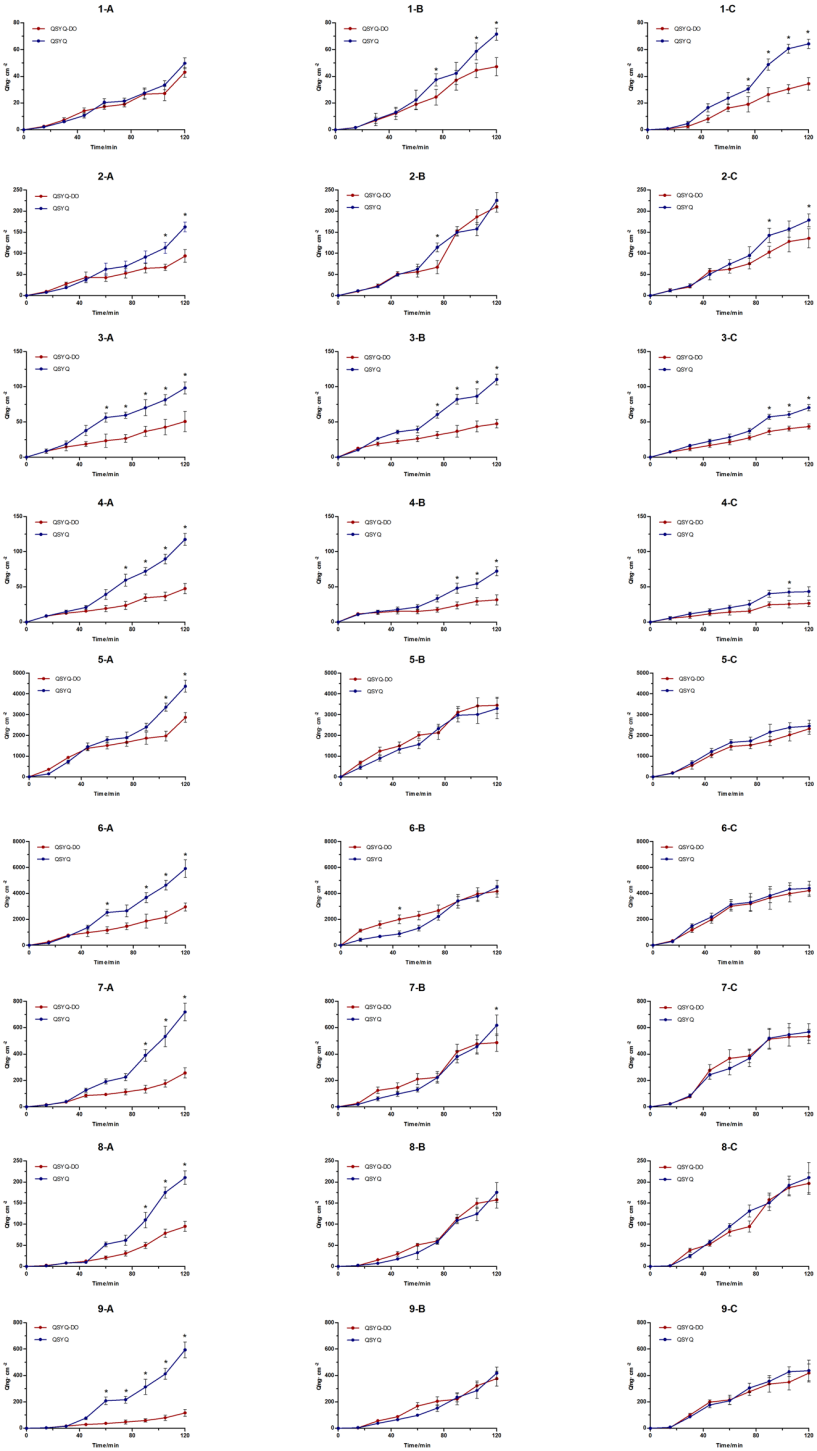
Accumulative absorption of major ingredients from QSYQ and QSYQ-DO by different intestinal segments. (**A**) Duodenum, (**B**) Jejunum, (**C**) Ileum. Compound label: 1. Formononetin, 2. Calycosin, 3. Ononin, 4. Calycosin-7-O-b-D-glycoside, 5. Protocatechuic aldehyde, 6. Tanshinol, 7. Rosmarinic acid, 8. Salvianolic acid B, and 9. Astragaloside IV. Data are expressed as mean ± SD, n = 3, *p < 0.05, **p < 0.01 *vs*. QSYQ-DO group.

In order to clarify the possible effect of DO volatile oils in QSYQ, contrastive absorption experiment was carried out. The intestinal absorptive behaviors of formononetin (FOR), calycosin (CS), ononin (ON), calycosin-7-O-β-D-glycoside (CG), protocatechuic aldehyde (PCA), tanshinol (TSL), rosmarinic acid (RA), salvianolic acid B (SAB) and astragaloside IV (AIV) in QSYQ and QSYQ-DO were simultaneously determined in the everted gut sacs model. As displayed in Fig. 4, time-dependent accumulation of tested compounds showed that in the presence of DO in QSYQ, the absorption of CS, ON, CG, PCA, TSL, RA, SAB, AIV in duodenum were tremendously enhanced. In the site of jejunum and ileum, the accumulation of FOR, ON, CG were obviously increased in QSYQ. It was obvious that compared to those of QSYQ-DO, the absorption of all tested compounds were increased, although in different extent at each time points, in the presence of DO, especially for ON, CG, RA, SAB and AIV. In contrast, nerolidol, the major component of DO volatile oils, was not detected in any group, indicating that the role of DO volatile oils is to facilitate the intestinal absorption of other major ingredients in QSYQ, not itself.

### QSYQ with DO volatile oils better attenuated DOX-induced myocardial injury and improved the cardiac function in mice

**Figure 5:**
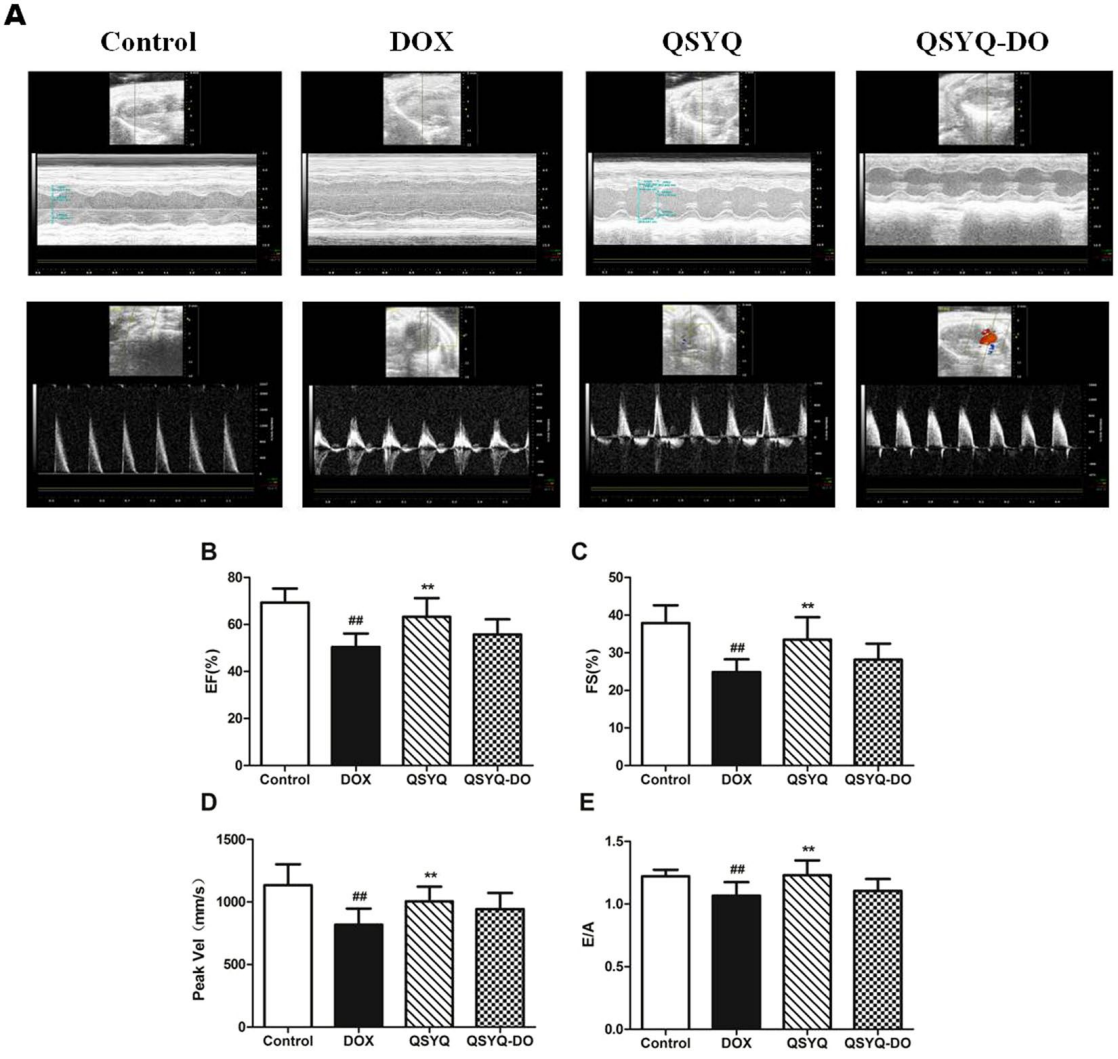
Effects of QSYQ and QSYQ-DO on mouse cardiac function. Left ventricular function measurements were assessed on echocardiography at the end of DOX injection. **(A)** Representative picture for M-mode, **(B)** Ejection fraction (EF%), **(C)** Fractional shortening (FS%), **(D)** Aortic valve peak velocity, and **(E)** Maximal early and late transmitral velocities (E/A). Data are expressed as mean ± SD, n = 8. ^##^p < 0.01 *vs*. Control group. **p < 0.01 *vs*. DOX group.

We further examined *in vivo* roles of QSYQ with or without DO volatile oils for myocardial injury protection in mice treated with DOX. As shown in Fig. 5 of the left ventricular function measurements assessed on echocardiography, ejection fraction (EF, Fig. 5B), fractional shortening (FS, Fig. 5C), peak velocity (Peak Vel, Fig. 5D) and E/A ratio (Fig. 5E) decreased significantly in the DOX group compared with the Control group. QSYQ pretreatment significantly improved the left ventricular function since there was a significant increase in EF, FS, peak vel and E/A ratio compared to the DOX group. In contrast, in QSYQ-DO group, these effects were not significant. These data suggest that the presence of DO volatile oils in QSYQ is critical in ameliorating DOX-induced cardiac dysfunction *in vivo* and its myocardial protection is superior to QSYQ-DO.

### QSYQ with DO volatile oils better decreased CK-MB, LDH release and inhibited myocardial apoptosis in mice

**Figure 6:**
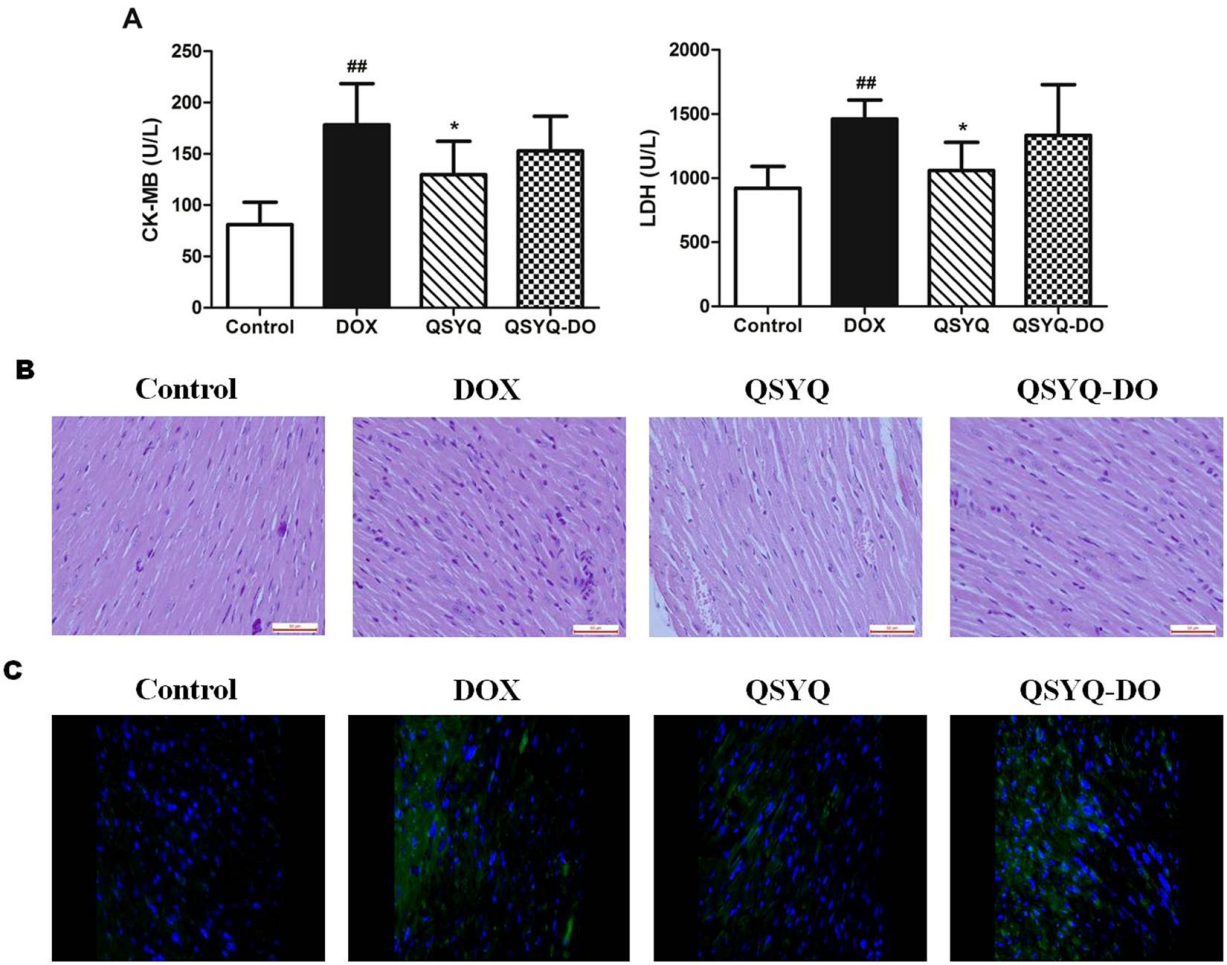
Effects of QSYQ and QSYQ-DO on cardiac enzyme release and myocardial histology. **(A)** CK-MB and LDH release in serum of DOX-treated mice, **(B)** Representative images showing H&E staining of heart sections (400 × magnification), and **(C)** Representative photomicrographs of TUNEL staining images. Total nuclei were labeled with DAPI (blue) and apoptotic nuclei were detected by TUNEL staining (green). One of 8 similar images was shown. All scale bars are 50 μm. Data are expressed as mean ± SD, n = 8. ^##^p < 0.01 *vs*. Control group. *p < 0.05 *vs*. DOX group.

Finally, to determine the consequences of QSYQ and QSYQ-DO pretreatment on DOX-induced myocardial injury, cardiac enzyme activities of CK-MB and LDH were determined. As shown in Fig. 6A, the levels of CK-MB and LDH were significantly increased in DOX group. Pretreatment with QSYQ significantly decreased CK-MB and LDH release whereas QSYQ-DO had no effect on these cardiac enzyme levels.

In order to directly observe the impact of QSYQ and QSYQ-DO pretreatment on DOX-induced alteration in myocardium structure, we examined the histology of the left ventricle myocardial tissues. As shown in Fig. 6B, distinct alterations, including rupture of myocardial fibers and infiltration of leukocytes, occurred in the myocardial tissues of the DOX group compared with the control group, which were ameliorated by pretreatment with QSYQ. Myocardial apoptosis is an essential contributor to cardiac dysfunction after DOX injury^20^. Therefore, terminal deoxynucleotidyl transferase-mediated deoxyuridine triphosphate nick-end labeling (TUNEL) staining was performed to explore whether QSYQ and QSYQ-DO prevented DOX-induced myocardial apoptosis. As expected, myocardial tissues from DOX-treated mice exhibited obvious apoptotic (TUNEL-positive) cells compared to control group (Fig. 6C). In contrast, a significantly lower proportion of apoptotic cells were observed in myocardial tissues from QSYQ-treated mice. However, in QSYQ-DO group, the effect was not significant. Taken together, these results confirmed that QSYQ inhibited myocardial apoptosis and protected the heart from DOX-induced injury, and its myocardial protection is superior to QSYQ-DO.

## Discussion

In summary, from the cell line level, QSYQ and QSYQ-DO both exerted considerable protective activities on mitochondrial dysfunction, no differences have been displayed. Interestingly, from isolated rat hearts to DOX-induced myocardial damage mice, QSYQ showed obvious superior pharmacological effect than QSYQ-DO, the variation in mice was even larger than that of isolated organs. In the view of results revealed by everted gut sac study, absorptive facilitate effect of DO volatile oils might closely related to the increased activities of QSYQ. On the other hand, DO volatile oils themselves could neither be absorbed across intestinal epithelial cells nor exerted significant pharmacological effects according to our study. Taking all the results into consider, it indicates that DO, as a courier herb component in QSYQ, does not directly contribute to the treatment effect of whole prescription, however, it facilitate the absorption of major active ingredients and increased their exposure, from this aspect, consequently enhance the potent of QSYQ, these findings might provide a solid foundation for better understanding the impacts of courier herb dedicating in complex CM prescriptions.

Certain pharmacological activities of DO have been reported^21^. Chemically, DO contains several sesquiterpenes, most of them have intensive volatilities^22^. In the CCM QSYQ, only extracted volatile oils from DO are used in a small quantity and a well-defined composition. Our previous study has established a gas chromatography (GC) quality control method for these volatile oils^23^, in this study, we developed the LC-MS method in order to determine and prove identical major ingredients from QSYQ and QSYQ-DO. Accordingly, nerolidol was used as an internal marker for DO volatile oils.

Oxidative stress is mainly caused by excessive accumulation of reactive oxygen species (ROS), which triggers the mitochondrial dysfunction and myocardial damage. It plays a central role in the pathogenesis of a number of cardiovascular diseases, such as ischemic heart disease, heart failure, and atherosclerosis^24, 25^. We adapted a model of H_2_O_2_-induced oxidative stress in H9c2 cardiomyocytes and found that H_2_O_2_ exposure led to significant injury in the cells, which was characterized by reduced cell viability and nuclear numbers (Supplementary Fig. S1C and Fig. 2A,B). Interestingly, the cardiomyocyte injury was significantly alleviated with both QSYQ and QSYQ-DO treatment. It is well established that the mitochondria play a key role in energy metabolism and biogenesis in cardiomyocytes^26^. Previous studies have demonstrated that damaged and dysregulated mitochondria generate redundant amounts of ROS, which leads to myocardial damage^27^. Mitochondrial dysfunction was also observed following H_2_O_2_ exposure, reflected by the loss of mitochondrial ΔΨm and intracellular ATP, and a rise in cytosolic Ca^2+^ preceded irreversible myocardial injury^25, 28^. Consistent with previously reported findings^14, 29^, QSYQ treatment significantly ameliorated the degree of mitochondrial damage. However, we found that this effect is independent of DO volatile oils since QYSQ-DO has the same effect in mitoprotection.

The recovery of cardiac function and myocardial infarct size have been considered as endpoints for ischemia/reperfusion injury evaluation in isolated rat hearts. Cardiodynamics, including HR, LVDP and ± dp/dtmax, alongside with I/R injury measurement, are considered as improvement parameters of left ventricular function in isolated rat hearts^30^. From this study, HR, LVDP and ± dp/dtmax at the end of reperfusion period were increased significantly by QSYQ pretreatment, which also showed an improved cardiac function and a decreased infarct size (Fig. 3). Meanwhile, we found that QSYQ-DO and DO volatile oils alone showed insignificant difference to I/R group on cardiac function and myocardial infarct size. These data suggested that QSYQ could ameliorate cardiac function after I/R injury and its cardioprotection was dependent on DO volatile oils since QSYQ-DO and DO volatile oils failed to do so in isolated rat hearts. This finding presented a clear contrast from the *in vitro* results in H9c2 cells where mitochondrial protection was equally executed by both QSYQ and QSYQ-DO, suggesting that one possible contribution of DO volatile oils in QSYQ is to facilitate its active ingredients reaching the target organ/tissues.

Intestine is a predominant absorptive site for oral medicines. Absorption and exposure of drug ingredients are critically influenced by their interactions with metabolism enzymes, transport proteins in intestinal epithelial cells, further causing different plasma concentrations and pharmacological diversities. Everted gut sac is an extensively used model for drug absorption evaluation and widely applied on herbal medicines^31–33^. Previous pharmacokinetic study has provided a hint that DO volatile oils mostly distributed and accumulated on rat intestine might affect the absorption of other drug ingredients^34^, but it has not been elucidated experimentally. In the present study, with the presence of DO volatile oils in QSYQ, the absorption of major ingredients in QSYQ, such as CS, ON, CG, PCA, TSL, RA, SAB, AIV were obviously enhanced compared to the absence of DO volatile oils in QSYQ-DO in isolated intestinal tract, especially for ON, CG, RA, SAB and AIV^35–38^, which have larger molecular weights and possess poor permeability across the intestinal epithelium.

Besides, from the viewpoint of chemical profiles, the major active chemical ingredients of QSYQ have been determined by previous investigations^39^, for example, CG, CS, ON and FOR are used as markers of isoflavonids. TSL, PCA, SAB and RA are proposed to be marker salvianolic acids, meanwhile, AIV is the presentative saponin in QSYQ. Moreover, the major ingredients we determined in everted gut sac experiment have been reported to be the marker components, as well as bioactive ingredients in QSYQ prescription^39, 40^. For example, CG, CS, FOR and RA possess considerable cardiomyocyte protect effects^41–43^, as well as CS and FOR are showed to be potent angiogenesis ingredients^44, 45^. TSL and PCA are reported to effectively alleviate myocardial ischemia/reperfusion injury and inhibit cardiomyocytes apoptosis^46^, while SAB greatly contribute to the anti-platelet aggregation and vasodilation effects of QSYQ^47, 48^. AIV could protect heart from ischemia and reperfusion injury via energy regulation mechanisms, and enhance the cardioprotective effects against acute myocardial infarction-induced heart failure and ventricular remodeling^49, 50^. Therefore, our finding that DO volatile oils preferentially enhanced absorption of these components is a strong validation for their documented effects on cardiac protection.

Drug-induced myocardial damage is an important risk factor for cardiomyopathy. Doxorubicin (DOX), one of the most common chemotherapy drugs, is well known for its cardiac toxicity^51^. Increasing studies have been published with respect to the mechanism responsible for the cardiac toxicity of DOX, suggesting that metabolic disorder may be a key factor in DOX induced cardiac injury^52^. In our study, DOX-induced decrease in cardiac function such as EF, FS, peak velocity and E/A ratio (Fig. 5), as well as increase in cardiac enzyme activities of CK-MB and LDH (Fig. 6) were all significantly prevented by pretreatment with QSYQ, suggesting a potential for QSYQ as a cardiac protection agent co-administrated with DOX for cancer chemotherapy. In the meantime, a significantly lower proportion of apoptotic cells was observed in heart slices from QSYQ-treated mice by TUNEL staining (Fig. 6). These results suggested that the potential of QSYQ to ameliorate DOX-induced disorders in cardiac structure and function and the probable mechanism might be the inhibition of myocardial cell apoptosis. Remarkably, QSYQ without DO volatile oils had no significant effects in protecting heart from DOX-induced injury, a stunning contrary from H_2_O_2_ induced cardiomyocyte injury *in vitro* and a nice confirmation for ischemia/reperfusion injury in isolated rat hearts.

In conclusion, we show that in a multi-component medicine, a component may be crucial only in an *in vivo* setting by maximizing other component’s pharmacological potentials. Accordingly, although DO volatile oils do not directly contribute to the myocardial protection, it facilitates the absorption of major active ingredients of QSYQ and increases their exposure, eventually enhancing the overall *in vivo* potency. Our findings may enhance our understanding of “courier herbs” in component-based Chinese medicine, with implications for better-designed “polypills” in future.

## Materials and Methods

### Materials

QiShenYiQi (QSYQ), QiShenYiQi without Dalbergia odorifera volatile oils (QSYQ-DO) and Dalbergia odorifera (DO) volatile oils were obtained from Tasly Pharmaceutical Group Co. Ltd. (Tianjin, China), which were prepared from water–ethanol extracts of Astragalus membranaceus, Salvia miltiorrhiza, Panax notoginseng and volatile oils by water extract and distilled from DO. The extracts of QSYQ, QSYQ-DO and DO volatile oils were dissolved in 10% ethanol to make stock solution at concentrations of 500 mg/mL. Doxorubicin hydrochloride for injection was purchased from Main Luck Pharmaceuticals Inc (Shenzhen, China). The kits for determination of creatine kinase-MB (CK-MB) and lactate dehydrogenase (LDH) were obtained from Jiancheng Bioengineering Institute (Nanjing, China). 2,3,5-triphenyl tetrazolium chloride (TTC) was purchased from Solarbio (Beijing, China). Dulbecco’s Modified Eagle’s Medium (DMEM) and other cell culture supplies were purchased from Gibco (Grand Island, NY, USA). Cell Count Kit-8 (CCK-8) was purchased from Dojindo (Kumamoto, Japan). Hydrogen peroxide (H_2_O_2_) was purchased from Sigma-Aldrich (St. Louis, MO USA). Hoechst 33342 and Mito Tracker Deep Red FM were obtained from Invitrogen (Eugene, USA), Rhod-2AM was from Dojindo (Kumamoto, Japan), Rhodamine 123 was obtained from Sigma (Santa Clara, USA). Fomic acid and acetonitrile used in UFLC-MS analysis were of HPLC grade. Water was purified using a Milli-Q water purification system (Milford, MA, USA). Krebs-Henseleit (K-H) solution contained 118.0 mM of sodium chloride, 4.69 mM of potassium chloride, 2.5 mM of calcium chloride, 1.18 mM of magnesium sulfate, 1.18 mM of potassium dihydrogen phosphate, 25.0 mM of sodium bicarbonate and 11.1 mM glucose. Chloramphenicol, diazepam, formononetin, calycosin, ononin, calycosin-7-O-β-D-glycoside, protocatechuic aldehyde, tanshinol, rosmarinic acid, salvianolic acid B and astragaloside IV were purchased from National Institutes for Food and Drug Control (Beijing, China).

### Animal and Ethics

C57BL/6 mice (18 ± 2 g) and Sprague Dawley rats (300 ± 20 g) were purchased from Beijing Vital River Lab Animal Technology Co. Ltd (Beijing, China). Ambient temperature (22 ± 2 °C) and relative humidity (60 ± 5%) were maintained. The animals were allowed to acclimatize to the housing facilities before the experiments and were provided with standard diet and water *ad libitum*. The animal experiments were performed in accordance with the recommendations in the Guidance for the Care and Use of Laboratory Animals issued by the Ministry of Science and Technology of China and were approved by the Laboratory Animal Ethics Committee of Tianjin University of Traditional Chinese Medicine (Permit Number: TCM-LAEC2016034).

### UFLC-MS analysis

Chromatography separation was performed on a Waters BEH C18 column (2.1 × 100 mm, 1.7μm) with a gradient elution of 0.05% fomic acid (A) and acetonitrile (B) as follows: 1% B from 0–0.5 min, 1–55% B from 0.5–5 min, 55–99% B from 5–8 min, 99% B maintained from 8–10 min at a flow rate of 0.4 mL/min. The column temperature was maintained at 20 °C and detection wavelength at 254 nm with injection volume of 2 μL for sample analysis.

Mass spectrum (MS) was performed on AB SCIEX Q-Trap 5500 triple quardruple mass spectrometry with electrospray ionization (ESI) interface. The MS spectra were acquired in both positive and negative modes with multiple reactions monitoring (MRM) mode for the analysis of main chemical components and internal standards (IS). In the negative ion mode, the MS conditions were as follows: ion source temperature, 550 °C; ion-spray voltage, −4500V; nebulizer gas and heater gas, 50 psi; curtain gas 25 psi. In positive ion mode, the ion-spray voltage was 5500 V and other parameters were the same as those in negative mode. Data acquisition and processing were performed by Analyst software (version 1.6.2).

### Cell culture

Rat embryonic ventricular myocardial cell line H9c2 was purchased from the Cell Bank of the Chinese Academy of Sciences (Shanghai, China). Cells were cultured in DMEM containing 4.5 g/L glucose supplemented with 10% fetal bovine serum (FBS) and 1% penicillin/streptomycin. All cells were maintained in a humidified incubator with 95% air/5% CO_2_ at 37 °C. The medium was replaced every 2–3 days, and the cells were subcultured or subjected to experimental procedures at 80–90% confluence. In all experiments, the cells were rendered quiescent by serum starvation for 24 h before treatment. After pretreatment with QSYQ, QSYQ-DO or DO volatile oils at 0.2 mg/mL for 24 h, the H9c2 cells were treated with 200 μM H_2_O_2_ for 2 h to induce cell injury.

### Assessment of cell viability with CCK-8 assay

Cell viability was assessed with the CCK-8 assay according to the manufacturer’s instructions. Briefly, H9c2 cells cultured in 96-well plates received the described treatments and the culture medium was replaced with 100 μL CCK-8 solution (DMEM:CCK-8 = 9:1), the plates were then incubated for a further 2 h. The absorbance was measured at 450 nm using a microplate reader (Flexstation 3, Molecular Devices, USA).

### Measurement of mitochondrial function with high content imaging system

For the detection of mitochondrial function, H9c2 cells were seeded in 96-well plate at a density of 5 × 10^3^ cells/well, and then pretreated with QSYQ, QSYQ-DO and DO volatile oils at 0.2 mg/mL for 24 h with or without 200 μM H_2_O_2_ for 2 h. After incubation, the cells were washed with PBS, and then incubated with 0.1 μg/mL Hoechst 33342, 0.1 μM Mito Tracker Deep Red FM, 3 μM Rhod-2AM and 3 μM Rhodamine 123 for 30 min at 37 °C. Then cells were washed twice with PBS and images were captured in Operetta High Content Imaging System (Perkin Elmer, Waltham, MA, USA) and quantification was done by the Harmony Software (Perkin Elmer) to determine mitochondrial function. Data were given as the nuclear number was analysed by Hoechst 33342, the intensity of Mito Tracker Deep Red FM (red fluorescence), Rhod-2AM (yellow fluorescence) and Rhodamine 123 (green fluorescence), indicating mitochondial mass and the levels of cytosolic Ca^2+^ and mitochondrial membrane potential (ΔΨ_m_).

### Isolated rat heart perfusion and Langendorff procedure

20 Rats were anesthetized with sodium pentobarbital (50 mg/kg i.p.). A thoracotomy was then performed and hearts were rapidly excised into an ice-cold K-H solution. After removal of the lungs and surrounding tissues, the aorta was attached to the perfusion device where hearts were perused at a constant retrograde flow, according to the method of Langendorff with a non-recirculated K-H solution, gassed with carbogen (95% O_2_ and 5% CO_2_) at 37 °C to obtain a physiological pH of 7.4. The hearts were perfused with constant pressure of approximately 65 mmHg and left for 20 min to permit recovery of function and stabilize the rhythm. The left ventricular end diastolic pressure was maintained at 5–10 mmHg, and cardiac function was evaluated by measuring left ventricular developed pressure (left ventricle end systolic pressure minus left ventricle end diastolic pressure; LVDP = LVSP − LVEDP), maximal and minimum rate of pressure development (±dp/dt_max_), and heart rate (HR). LVDP, ±dp/dt_max_, and HR were calculated from the left ventricular pressure curve. These parameters were recorded continuously on a computer using Powerlab data acquisition system (8SP Chart 7 software; A.D. Instruments, Castle Hill, Australia)^53^.

Hearts were then randomly assigned to the following groups: (1) Control group (hearts perfused with K-H buffer for 120 min); (2) I/R group (hearts that were allowed to stabilize for 30 min prior to being subjected to 30 min global ischemia achieved by discontinued K-H buffer perfusion, followed by 60 min reperfusion); (3) QSYQ group (hearts perfused with QSYQ 0.2 mg/mL for 10 min after 20 min of stabilization, global ischemia for 30 min, followed by 60 min reperfusion); (4) QSYQ-DO group (hearts perfused with QSYQ-DO 0.2 mg/mL for 10 min after 20 min of stabilization, global ischemia performed for 30 min, followed by 60 min reperfusion) and DO group (hearts perfused with DO volatile oils 0.2 mg/mL for 10 min after 20 min of stabilization, global ischemia performed for 30 min, followed by 60 min reperfusion).

### Determination of myocardial infarction size

After 60 min reperfusion, hearts were removed from Langendorff apparatus, aortic root excised and kept in −20 °C for 30 min. Frozen hearts were cut transversely into different slices like 2–3 mm which were incubated in 1% 2,3,5-triphenyl tetrazolium chloride (TTC) dissolved in 0.1 M phosphate buffer (pH 7.4) at 37 °C for 15 min. The infracted areas were displayed as the area unstained by TTC^54^. The slices were then photographed for further analysis. The extents of the necrosed areas were quantified by Image J software. Total area of infarct and risk areas were then calculated and expressed as a percentage of the whole heart area.

### Rat everted gut sac experiment

Everted gut sac experiment was performed according to previously published procedures^55^. Briefly, 6 rats were randomly divided into QSYQ and QSYQ-DO groups, starved for 12 hours before experiment and euthanized by cervical dislocation, and the duodenum, jejunum and ileum were immediately removed and flushed by saline respectively. The intestinal segments were instantly cut into small units with approximate 10 cm and everted quickly, then put into oxygenated K-H solution (5%CO_2_ + 95% O_2_). One end of the intestinal segments was tied with suture and the other end was immobilized by a plastic tube in order to generate a sampling site. Then the segments were filled with 1 mL K-H solution and put into containers with 40 mL QSYQ K-H solution or QSYQ-DO K-H solution at 37 °C with the same concentration of 10 mg/mL. 200 uL solution inside the segments was collected at 15, 30, 45, 60, 90, 105 and 120 min and compensated with an equal volume of K-H solution. All the samples were analyzed using UFLC-MS method. After analysis, the accumulate amounts (ng/cm^2^) of absorbed formononetin (FOR), calycosin (CS), ononin (ON), calycosin-7-O-β-D-glycoside (CG), protocatechuic aldehyde (PCA), tanshinol (TSL), rosmarinic acid (RA), salvianolic acid B (SAB) and astragaloside IV (AIV) were calculated. The result data were presented by time-accumulative curves of absorption amount.

### Animal model and drug administration in mice

C57BL/6 mice were randomly assigned to 5 treatment groups, with n = 8 in each group. Doxorubicin (DOX) dissolved in saline were given by tail vein injection (once a week × 3) to reach a cumulative dose of 15 mg/kg (DOX group) or volume-matched saline (Control group). Other groups of mice received QSYQ or QSYQ-DO (both dissolved in saline at dose of 5 mg/mL) by intragastrical administration for 15 days before DOX injection (QSYQ group and QSYQ-DO group). Body weights were measured every 3 days in each group.

### Echocardiographic assessment of cardiac function

Echocardiography was performed by a researcher blinded to the experiment. Mice with or without 15-days of QSYQ or QSYQ-DO pretreatment before exposure to DOX were lightly anaesthetized with inhaled isoflurane (2% vaporized with 100% oxygen). Long-axis view was obtained using an ultrahigh resolution small animal ultrasound imaging system (Vevo2100, Visual Sonics, Canada) with a 30 MHz linear array transducer (MS 400). Respiration tracing was synchronized with imaging, and examinations lasted for 30 min. Functional indicators such as Left ventricular end-systolic and end-diastolic diameters were used to determine left ventricular ejection fraction (EF) and fractional shortening (FS), which were considered as an important indicators of left ventricular contractile function. Pulsed wave Doppler measurements were obtained in the apical view with a cursor at mitral valve inflow for maximal early (E) and late (A) transmitral velocities in diastole. The aortic valve blood flow was assessed by Doppler measurement under a modified view.

### Creatine kinase-MB and lactate dehydrogenase assay

At the end of the cardiac ultrasound, mice were sacrificed under anesthesia. Blood was collected and centrifuged at 1,000 rpm for 10 minutes. Serum was used to assess the content of creatine kinase-MB (CK-MB) and lactate dehydrogenase (LDH) by automatic biochemical detector (MK3; Thermo Fisher Scientific, Waltham, MA, USA) according to the manufacturer’s instruction. CK-MB and LDH levels were expressed in units per liter of serum.

### Histological analysis

After collecting the blood samples, hearts were removed. Heart tissues (n = 3) were fixed in 4% paraformaldehyde (PFA) solution for more than 48 h, and further prepared for paraffin sectioning. Serial sections (4 μm thick) were cut and stained with hematoxylin and eosin (H&E) for morphological analysis under light microscope. Photographs of left ventricular sections cut from the same location of each heart were observed under ×400 magnification by a digital camera connected to an optical microscope (DM3000, Leica Microsystems, Wetzlar, Germany) and processed with a Leica Application Suite (Leica Microsystems, Wetzlar, Germany).

Paraffin heart sections were deparaffinized in xylene and prepared for immunohistological staining. Terminal deoxynucleotidyl transferase-mediated deoxyuridine triphosphate nick-end labeling (TUNEL) staining was carried out to assess myocardial apoptosis using the Fluorescein *In Situ* Cell Death Detection Kit (Roche Diagnostics, Indianapolis, IN) as previously described^56^. Apoptotic nuclei and total cardiomyocyte nuclei were labeled with green fluorescein staining and DAPI, respectively. For each slice, 5 fields were randomly obtained under a confocal scanning microscope (DP71, Olympus, Japan). Extent of cell apoptosis was expressed as a ratio of TUNEL positive nuclei over DAPI-stained nuclei.

### Statistical analysis

All values are expressed as the mean ± SD. Comparisons between multiple-group means were performed using one-way analysis of variance (one-way ANOVA). Multiple comparisons between the groups were performed using LSD method. *P* values < 0.05 were considered to be statistically significant. All statistical analyses were performed using SPSS version 17.0.

## Electronic supplementary material

Supplementary Information

## References

[1] Muscente F, De Caterina R (2016). Polypill: quo vadis?. J Cardiovasc Med (Hagerstown).

[2] Castellano JM, Sanz G, Penalvo JL (2014). A polypill strategy to improve adherence: results from the FOCUS project. Journal of the American College of Cardiology.

[3] Ferreira AS, Lopes AJ (2011). Chinese medicine pattern differentiation and its implications for clinical practice. Chinese journal of integrative medicine.

[4] Wang Y, Liu Z, Li C (2012). Drug target prediction based on the herbs components: the study on the multitargets pharmacological mechanism of qishenkeli acting on the coronary heart disease. Evidence-based complementary and alternative medicine: eCAM.

[5] Han, J. Y., Li, Q., Ma, Z. Z. *et al*. Effects and mechanisms of compound Chinese medicine and major ingredients on microcirculatory dysfunction and organ injury induced by ischemia/reperfusion. *Pharmacology & therapeutics* (2017). 10.1016/j.pharmthera.2017.03.005 28322971

[6] Zhang Boli WY (2006). Shang Hongcai Theories and Methods Used in the Research of Modern Chinese Medicine by Drug Combination. Continuing Medical Education.

[7] Zhang JH, Zhu Y, Fan XH (2015). Efficacy-oriented compatibility for component-based Chinese medicine. Acta pharmacologica Sinica.

[8] Wang L, Zhou GB, Liu P (2008). Dissection of mechanisms of Chinese medicinal formula Realgar-Indigo naturalis as an effective treatment for promyelocytic leukemia. Proceedings of the National Academy of Sciences of the United States of America.

[9] Tang DX, Zhao HP, Pan CS (2013). QiShenYiQi Pills, a Compound Chinese Medicine, Ameliorates Doxorubicin-Induced Myocardial Structure Damage and Cardiac Dysfunction in Rats. Evidence-based complementary and alternative medicine: eCAM.

[10] JianXin C, Xue X, ZhongFeng L (2016). Qishen Yiqi Drop Pill improves cardiac function after myocardial ischemia. Scientific reports.

[11] Wang J, Lu L, Wang Y (2015). Qishenyiqi Dropping Pill attenuates myocardial fibrosis in rats by inhibiting RAAS-mediated arachidonic acid inflammation. Journal of ethnopharmacology.

[12] Chen YY, Li Q, Pan CS (2015). QiShenYiQi Pills, a compound in Chinese medicine, protects against pressure overload-induced cardiac hypertrophy through a multi-component and multi-target mode. Scientific reports.

[13] Wang Y, Wang J, Guo L (2012). Antiplatelet effects of qishen yiqi dropping pill in platelets aggregation in hyperlipidemic rabbits. Evidence-based complementary and alternative medicine: eCAM.

[14] Chen JR, Wei J, Wang LY (2015). Cardioprotection against ischemia/reperfusion injury by QiShenYiQi Pill(R) via ameliorate of multiple mitochondrial dysfunctions. Drug design, development and therapy.

[15] Wu L, Wang Y, Li Z (2014). Identifying roles of “Jun-Chen-Zuo-Shi” component herbs of QiShenYiQi formula in treating acute myocardial ischemia by network pharmacology. Chinese medicine.

[16] Li X, Wu L, Liu W (2014). A network pharmacology study of Chinese medicine QiShenYiQi to reveal its underlying multi-compound, multi-target, multi-pathway mode of action. PloS one.

[17] Zheng S, Zhang Y, Qiao Y (2015). The Mechanism Research of Qishen Yiqi Formula by Module-Network Analysis. Evidence-based complementary and alternative medicine: eCAM.

[18] Yang ZH, Mei C, He XH (2013). [Advance in studies on chemical constitutions, pharmacological mechanism and pharmacokinetic profile of dalbergiae odoriferae lignum]. Zhongguo Zhong yao za zhi=Zhongguo zhongyao zazhi=China journal of Chinese materia medica.

[19] Zheng X, Zhao X, Wang S (2007). Co-administration of Dalbergia odorifera increased bioavailability of Salvia miltiorrhizae in rabbits. The American journal of Chinese medicine.

[20] Zhu SG, Kukreja RC, Das A (2011). Dietary nitrate supplementation protects against Doxorubicin-induced cardiomyopathy by improving mitochondrial function. Journal of the American College of Cardiology.

[21] Ham, S. A., Kang E. S., Yoo, T. *et al*. Dalbergia odorifera Extract Ameliorates UVB-Induced Wrinkle Formation by Modulating Expression of Extracellular Matrix Proteins. *Drug development research* (2015). 10.1002/ddr.21240 25620496

[22] Tao Y, Wang Y (2010). Bioactive sesquiterpenes isolated from the essential oil of Dalbergia odorifera T. Chen. Fitoterapia.

[23] Li Ming LR-m ZY-qian, Shi JW HY, G C (2016). Fingerprint of volatile components in Qishen Yiqi Dropping Pills. Chinese Traditional and Herbal Drugs.

[24] Marzetti E, Csiszar A, Dutta D (2013). Role of mitochondrial dysfunction and altered autophagy in cardiovascular aging and disease: from mechanisms to therapeutics. American journal of physiology Heart and circulatory physiology.

[25] Akao M, O’Rourke B, Teshima Y (2003). Mechanistically distinct steps in the mitochondrial death pathway triggered by oxidative stress in cardiac myocytes. Circulation research.

[26] Ren J, Pulakat L, Whaley-Connell A (2010). Mitochondrial biogenesis in the metabolic syndrome and cardiovascular disease. J Mol Med (Berl).

[27] Steinberg SF (2013). Oxidative stress and sarcomeric proteins. Circulation research.

[28] Mao CY, Lu HB, Kong N (2014). Levocarnitine protects H9c2 rat cardiomyocytes from H2O2-induced mitochondrial dysfunction and apoptosis. International journal of medical sciences.

[29] Lin SQ, Wei XH, Huang P (2013). QiShenYiQi Pills(R) prevent cardiac ischemia-reperfusion injury via energy modulation. International journal of cardiology.

[30] Yu J, Wang L, Akinyi M (2015). Danshensu protects isolated heart against ischemia reperfusion injury through activation of Akt/ERK1/2/Nrf2 signaling. International journal of clinical and experimental medicine.

[31] Li M, Si L, Pan H (2011). Excipients enhance intestinal absorption of ganciclovir by P-gp inhibition: assessed *in vitro* by everted gut sac and *in situ* by improved intestinal perfusion. International journal of pharmaceutics.

[32] Chula S, Hang L, Yinying B (2012). The effects of notoginsenoside R(1) on the intestinal absorption of geniposide by the everted rat gut sac model. Journal of ethnopharmacology.

[33] Xin Y, Liu S (2015). Quantitative Assessment of the Influence of Rhizoma Zingiberis on the Level of Aconitine in Rat Gut Sacs and Qualitative Analysis of the Major Influencing Components of Rhizoma Zingiberis on Aconitine Using UPLC/MS. PloS one.

[34] Zhang Y, Shi P, Yao H (2012). Metabolite profiling and pharmacokinetics of herbal compounds following oral administration of a cardiovascular multi-herb medicine (Qishen yiqi pills) in rats. Current drug metabolism.

[35] Gu Y, Wang G, Pan G (2004). Transport and bioavailability studies of astragaloside IV, an active ingredient in Radix Astragali. Basic & clinical pharmacology & toxicology.

[36] Xu F, Zhang Y, Xiao S (2006). Absorption and metabolism of Astragali radix decoction: in silico, *in vitro*, and a case study *in vivo*. Drug metabolism and disposition: the biological fate of chemicals.

[37] Konishi Y, Kobayashi S (2005). Transepithelial transport of rosmarinic acid in intestinal Caco-2 cell monolayers. Bioscience, biotechnology, and biochemistry.

[38] Jin X, Zhang SB, Li SM (2016). Influence of Chitosan Nanoparticles as the Absorption Enhancers on Salvianolic acid B *In vitro* and *In vivo* Evaluation. Pharmacognosy magazine.

[39] Jing Wang NW (2010). Global Chemome Study by LC Coupled with DAD and ESI–Q–TOF MS of a Composite Traditional Chinese Medicine Qishenyiqi Dropping Pills. Chromatographia.

[40] Yunfei LYW, Qu H, Cheng Y (2008). Simultaneous Determination of Seven Bioactive Compounds in Chinese Medicine “QI-SHEN-YI-QI” Dropping Pill by LC-UV and LC-ELSD. Chromatographia.

[41] Jiang YH, Sun W, Li W (2015). Calycosin-7-O-beta-D-glucoside promotes oxidative stress-induced cytoskeleton reorganization through integrin-linked kinase signaling pathway in vascular endothelial cells. BMC complementary and alternative medicine.

[42] Liu B, Zhang J, Liu W (2016). Calycosin inhibits oxidative stress-induced cardiomyocyte apoptosis via activating estrogen receptor-alpha/beta. Bioorganic & medicinal chemistry letters.

[43] Diao J, Wei J, Yan R (2016). Rosmarinic Acid suppressed high glucose-induced apoptosis in H9c2 cells by ameliorating the mitochondrial function and activating STAT3. Biochemical and biophysical research communications.

[44] Tang JY, Li S, Li ZH (2010). Calycosin promotes angiogenesis involving estrogen receptor and mitogen-activated protein kinase (MAPK) signaling pathway in zebrafish and HUVEC. PloS one.

[45] Huh JE, Kwon NH, Baek YH (2009). Formononetin promotes early fracture healing through stimulating angiogenesis by up-regulating VEGFR-2/Flk-1 in a rat fracture model. International immunopharmacology.

[46] Wei G, Guan Y, Yin Y (2013). Anti-inflammatory effect of protocatechuic aldehyde on myocardial ischemia/reperfusion injury *in vivo* and *in vitro*. Inflammation.

[47] Shou Q, Pan Y, Xu X (2012). Salvianolic acid B possesses vasodilation potential through NO and its related signals in rabbit thoracic aortic rings. European journal of pharmacology.

[48] Wu YP, Zhao XM, Pan SD (2008). Salvianolic acid B inhibits platelet adhesion under conditions of flow by a mechanism involving the collagen receptor alpha2beta1. Thrombosis research.

[49] Cheng S, Yu P, Yang L (2016). Astragaloside IV enhances cardioprotection of remote ischemic conditioning after acute myocardial infarction in rats. American journal of translational research.

[50] Tu L, Pan CS, Wei XH (2013). Astragaloside IV protects heart from ischemia and reperfusion injury via energy regulation mechanisms. Microcirculation.

[51] Monsuez JJ, Charniot JC, Vignat N (2010). Cardiac side-effects of cancer chemotherapy. International journal of cardiology.

[52] Green PS, Leeuwenburgh C (2002). Mitochondrial dysfunction is an early indicator of doxorubicin-induced apoptosis. Biochimica et biophysica acta.

[53] Fan G, Yu J, Asare PF (2016). Danshensu alleviates cardiac ischaemia/reperfusion injury by inhibiting autophagy and apoptosis via activation of mTOR signalling. Journal of cellular and molecular medicine.

[54] Ito WD, Schaarschmidt S, Klask R (1997). Infarct size measurement by triphenyltetrazolium chloride staining versus *in vivo* injection of propidium iodide. Journal of molecular and cellular cardiology.

[55] Barthe L, Woodley JF, Kenworthy S (1998). An improved everted gut sac as a simple and accurate technique to measure paracellular transport across the small intestine. European journal of drug metabolism and pharmacokinetics.

[56] Zhao Y, Xu L, Qiao Z (2016). YiXin-Shu, a ShengMai-San-based traditional Chinese medicine formula, attenuates myocardial ischemia/reperfusion injury by suppressing mitochondrial mediated apoptosis and upregulating liver-X-receptor alpha. Scientific reports.

