# Supplementary material for: A critical courier role of volatile oils from Dalbergia odorifera for cardiac protection in vivo by QiShenYiQi
Source: Sci Rep. 2017 Aug 4;7:7353. doi: 10.1038/s41598-017-07659-x (PMC5544742; doi:10.1038/s41598-017-07659-x)
Supplement: Supplementary file 1 — Supplementary Information [file 41598_2017_7659_MOESM1_ESM.pdf]

A critical courier role of volatile oils from Dalbergia odorifera for cardiac protection *in vivo* by QiShenYiQi

Jiahui Yu[1, 2#], Wen Zhang[1, 3#], Yiqian Zhang[3, 4], Yadong Wang[1], Boli Zhang[1], Guanwei Fan[1, 5*], Yan Zhu[1, 2*]

[1]Tianjin State Key Laboratory of Modern Chinese Medicine, Tianjin University of Traditional Chinese Medicine, Tianjin, China

[2]Research and Development Center of TCM, Tianjin International Joint Academy of Biotechnology & Medicine, Tianjin, China

[3]State Key Laboratory of Core Technology in Innovative Chinese Medicine, Tianjin Tasly Holding Group Co., Ltd., Tianjin, China

[4]State Key Laboratory of Natural Medicines, China Pharmaceutical University, Nanjing, China

[5]The First Teaching Hospital of Tianjin University of Traditional Chinese Medicine Tianjin, China,

[#] Equal contribution to this work

[*] Correspondence should be addressed to Yan Zhu, yanzhu.harvard@iCloud.com (YZ) and Guanwei Fan, fgw1005@hotmail.com (GWF)

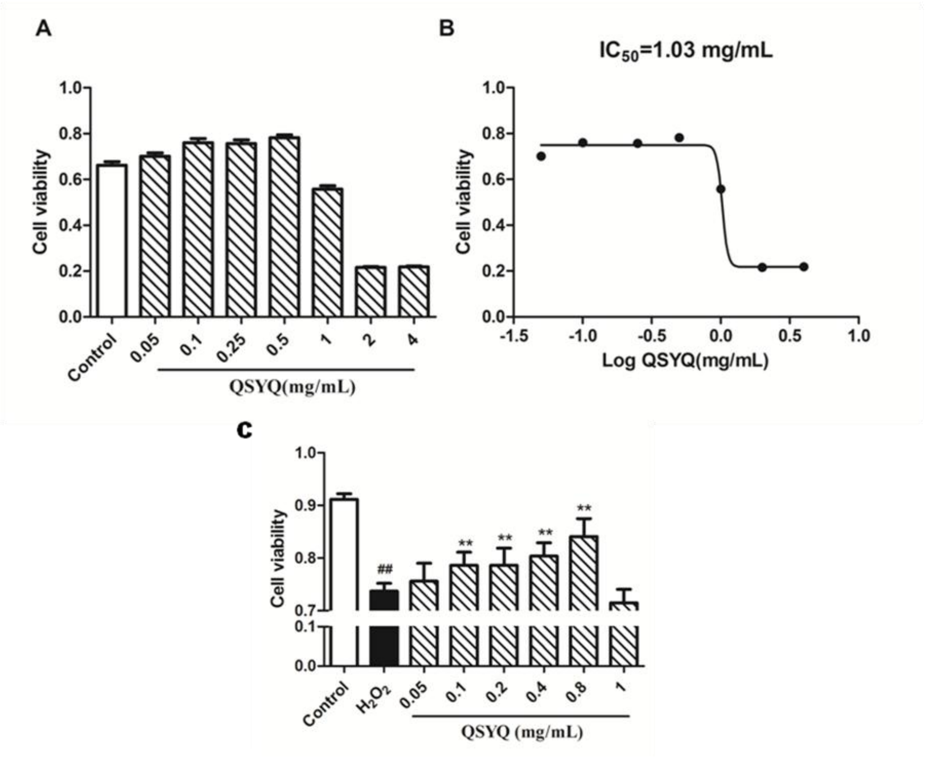

Supplementary Fig S1. Determination of *in vitro* QSYQ dose by cell viability assay. (A) Toxicity concentration of QSYQ to untreated cells. (B) IC_{50} curve of QSYQ. (C) QSYQ increased cell viability in concentration-dependent manner. Data are expressed as the mean±SD, n=3. $^{##}p<0.01$ *vs*. Control group. $^{**}p<0.01$ *vs*. H_2O_2 group.
